# The Influence of Oxyphosphate of Zinc Cements on the Tooth Tissues

**Published:** 1900-04-15

**Authors:** N. S. Hoff


### DENTAL REGISTER.

Vol. LIV. APRIL 15, 1900. No. 4.

### THE INFLUENCE OF OXYPHOSPHATE OF ZINC CEMENTS ON THE TOOTH TISSUES.

BY N. S. HOFF, D.D S.

Read before the Detroit Dental Society, November 13, 1899.

The liquid of oxyphosphates is glacial phosphoric acid, and is manufactured in Germany only for commercial purposes. It is rarely a pure article, and cement manufacturers claim that this accounts for the varieties and eccentricities encountered in the use of this material for fillings.

By the chemist this acid is made from phosphoric oxide, or anhydrous phosphoric acid, which is obtained by burning phosphorus in dry oxygen gas. This oxide is a white powder, deliquescent, and volatile at a red heat; when fused and allowed to cool it assumes a vitreous appearance. By dissolving this anhydrous acid in water at different temperatures three different phosphoric acids are obtained, the meta-phosphoric or monobasic is the glacial acid used in making oxyphosphate cements; its chemical formula is $H_2O,P_2O_5$ or, hydrated, $HPO_3$.

This acid differs from the other two in that it coagulates albumen and precipitates solutions of baryta, lime and silver, which the others do not.

Much of the commercial glacial phosphoric acid is obtained by dissolving calcined bones in sulphuric acid securing an acid phosphate of calcium, which is mixed with carbonate of ammonium and burned to glacial phosphoric acid. It is a white, uncrystallizable solid; fusible, inodorous, very sour and slowly soluble in water and alcohol.

Most commercial glacial phosphoric acid is impure, containing varying but large percentages of sodium phosphate, rarely less than 55 percent. It is also liable to contain arsenic, ammonium and magnesium. The sodium phosphate, if not in excess, is probably not harmful, as it only modifies the setting of the cement, makes it set slower.

The powder used ordinarily is the oxide of zinc ($ZnO$). Official oxide of zinc is a nearly white, odorless and tasteless powder, insoluble in water or alcohol; it may be contaminated with iron, lead, silica, arsenic and tin. It is said the whiter preparations are more likely to be impure than those with a yellowish tint. The yellow color of oxyphosphate powder is artificially produced with the phosphate of silver. Flagg condemns commercial oxide of zinc as an ingredient of cements for fillings, because of these many impurities, and recommends that the oxide be treated with nitric acid, then dried, calcined and pulverized; by this means a powder is obtained distinguished for dryness and quick fineness, but slightly gritty and heavier than commercial oxide of zinc. The mixing of the acid and powder in proper proportions and in accordance with well-known manipulative procedures, will result in a hard and comparatively insoluble cement. Andreae, according to Dr. Miller, suggests that 196 parts, by weight, of the acid be used with 243 parts of the powder (about 4 to 5).

This makes a thin mix such as would be used in capping a pulp, and on theoretical principles should produce, when accurately manipulated, an oxyphosphate cement with no excess of either chemical agent. But practically, for reasons already indicated in the comments on the materials and because of faulty manipulation, we are not likely to obtain this precision either in the products at command or their manipulation. This, in passing, should not, however, deter us from aiming at scientific accuracy, even though we may not hope to attain it.

Dr. Miller says in an article on this material in *Cosmos*, 1891, page 17: "We are obliged to incorporate enough of the powder into the liquid to obtain a paste of 'the proper consistency,' without regard to the chemical aspect of the question. Whether we add more or less of the powder than is demanded by the chemical formula, I am unable to say. I have noticed, however, that the paste invariably has a decided acid reaction, due to the presence of free acids (or acid salts). Not only is this true of the freshly mixed paste, but if we moisten a piece of cement which has already become perfectly hard, we shall find that it readily gives up acid. Break up a ball of cement as large as a pea into small pieces and put them into a test tube, and cover them with two or three drops of water; test this water with blue litmus paper, and it will be found to have a more or less pronounced acid reaction. Furthermore, thin pieces of dentin dropped into it will show signs of decalcification in four to six days. This reaction will vary with the different preparations of the phosphate cements."

To confirm these experiments I made a 5 percent solution of egg albumen and dipped into it slips of litmus paper which gave decided alkaline reaction. These slips while still wet were lain on a clean glass slab, and cement fillings mixed in varying consistencies and allowed to set were dropped onto the moist litmus paper; every test showed the characteristic acid reaction by a red circle under and in the immediate vicinity of the cement. I found the harder and older cement mixes produced only slightly less reaction than the newer mixes. Two well mixed and hard masses weighing about one grain each were placed in one-half dram of the albumen solution, and after twenty-four hours they had imparted no appreciable acid reaction to it. One of the cement masses was then pulverized in a mortar and placed in a small test tube and ten drops of the albumen solution added and immediately tested for acid, with very positive indications. Ten drops more of the albumen solution was added and the test showed a slightly alkaline color. This experimentation would seem to indicate that there was sufficient free acid in an ordinary pulp capping, even when thoroughly crystallized, to at least change, chemically, the normal serum of a pulp.

Further, Dr. Miller in his article relates two other experiments of interest in this connection. He bored a number of shallow holes into a piece of ivory, some of which he filled with oxyphosphate and others with gutta-percha. The block of ivory was then inverted and placed, fillings down, on a piece of moist, bibulous paper, in the bottom of a shallow glass vessel, which was securely covered to prevent evaporation. After several days the fillings were removed and the cavities washed out, and the ivory stained with fuchsine. It was found that the walls of the cavities which had been filled with cement took the stain more profoundly than those which had contained the gutta-percha, which would seem to indicate disintegration at least, if not actual decalcification.

Again he bored a shallow hole into a piece of moist bone and introduced a minute quantity of lactic acid, with intent to produce a thin layer of decalcified bone at the bottom of the cavity. In fifteen minutes the cavity was washed out, neutralized and filled with oxyphosphate cement. It was then rolled in wet bibulous paper and left for twelve hours. The filling was removed and the softened bone at the bottom of the cavity tested and found to have a decided acid reaction, indicating clearly that the cement had parted with a portion of its acid.

No systematic research has been made to determine whether the oxyphosphate cements have a tendency to devitalize pulps when placed in contact with them. But there are frequent references in our literature to the probable action of oxyphosphate fillings in destroying pulps. Burchard, in his text-book, page 573, says: "Oxide of zinc may contain arsenic trioxide as an impurity, in which event cements made of it may kill the dental pulp." Dr. Niles, in *Cosmos*, 1891, page 248, says: "A given amount of acid can only unite with a quantity of powder sufficient to satisfy its affinity; if there be an excess of powder the mix is brittle and crumbly, but an excess of acid makes a dangerous substance, destructive to the tooth."

Dr. Miller relates a case, *Cosmos*, page 18, of a lad of twelve years, who had cavities in the proximal surfaces of all the upper incisor teeth. The teeth were soft but none of the pulps were exposed. The cavities were all filled with cement. A little more than a year later the pulps in all four incisors were dead.

Many other recorded instances of possible deleterious action of this cement on pulps might be cited, but these will serve to justify your committee for placing this subject on your program and inviting your discussion. To enable us to conduct our discussion of it within the compass of time allowed to us this evening, I will suggest that it must be due to one of two causes: first, imperfect manipulation of the material, and second, to the inherent qualities of the material itself. I shall not consume much of your time in discussing the first cause, for the reason that I am not bold enough to even insinuate that any one present has not sufficient skill to manipulate oxyphosphate of zinc as scientifically and skillfully as myself. I will simply suggest that good and pure material may be spoiled by improper manipulation, as may be inferred from remarks made in connection with our examination of the material itself. Neither do I care to say that any one who fails to get good results from its use does not know how to skillfully adapt the material without causing mechanical irritation which may prove fatal, and yet this is not an unimportant element in its successful use. We will concede, if you please, that the fault is not, or ought not at least, to be looked for in the direction of faulty manipulation or lack of essential precautions, and pass to a brief consideration of the material itself.

The U. S. Dispensatory tells us that zinc oxide is a white powder, permanent in the air, odorless, tasteless, insoluble in water or alcohol, but soluble in acids. It is used in medicine as a tonic, anti-spasmodic and astringent; externally, as an excitant to excoriated surfaces. It has the great advantage over preparations of lead in being non-toxic. The internal dose is from two to eight grains or more, repeated several times a day. All of which would indicate that it was not at all a dangerously destructive drug, used locally, in limited quantities. When applied to sensitive, soft tissues it is not escharotic or caustic, only slightly irritant and astringent. And because of its comparative insolubility, it can not possibly be poisonous in the limited quantity used in capping an exposed pulp.

To the point raised by Dr. Burchard, that the destructive action of oxyphosphate cements was due to the presence of arsenic in the oxide of zinc, I would say that, while this is possible, I can not conceive of there being sufficient arsenic in the small amount of cement used in this way to produce so extensive a result. The percentage of arsenic in any case, even with a very crude material, would be small and when disseminated through the insoluble cement and securely fixed there would be slight chance for its activity, even should the pulp exposure be quite extensive. And, similarly, I can not conceive of the small amounts of other possible impurities in cements having a like deleterious action. And to further uphold my position, I take for granted that we are careful to obtain from the most reliable makers our supplies, and consequently we are not likely to have such contingencies to contend with.

If then it can not be the oxide of zinc which is at fault, what can we say about the probable physiological action of the other ingredient of our cement which might produce this destructive action?

The glacial phosphoric acid, as we have already seen, is likely to be impure, or at least contain substances not necessary to usefulness in making cements. Commercial glacial acid is very sour to the taste and astringent, it is also irritant and will coagulate albumen, and when confined in a local application for a time it produces destruction of the soft tissues and a sloughing, showing that it has a cauterant tendency when used in this way. It is claimed that pyrophosphoric acid and orthophosphoric acid ($2H_2O,P_2O_5$ and $3H_2O,P_2O_5$) are not cauterant. I have found these latter acids do not coagulate albumen, even when equal quantities of the 5 percent solution of albumen and the acids are mixed at ordinary temperatures, but when heated they seem to increase the facility with which coagulation is reached. All are undoubtedly irritant in the uncombined state, and even when mixed with the inert oxide of zinc they produce excessive pain when applied to sensitive dentin, not wholly due to the irritant action of the acid, however, as the difference in temperature and chemical reaction which results and is but temporary in results, has much to do with the pain produced.

The experiments of Prof. Miller would seem to indicate conclusively that either the acid or its combination with the oxide of zinc produces more than a passing irritant action. The other experiments to which I have referred also suggest that this corrosive action, which Miller observed, is due to the free acid existing in the cement, because used in excess, or set free by the solvent action of natural solvents in the tooth filled by the cement. We are led to conclude, therefore, that oxyphosphate pulp cappings may endanger the vitality of the pulp, especially when there are large exposures, the pulp of low grade in vitality, and where cements are employed carelessly, or when the ingredients of the cement are not chemically pure.

Another point which ought to be carefully noted in this connection, is that the oxyphosphate cements have no sterilizing or antiseptic action.

Prof. Miller, in *Cosmos*, 1889, page 920, conclusively demonstrates this by a series of experiments made to determine the relative antiseptic value of the various filling materials, so that its use without some precedent or accompanying antiseptic might leave the way open for the destructive action of the ever-present micro-organism. This is especially important since the result of bacterial action is very often the liberation of ammonia, the great foe of oxyphosphate cements.

Again, while the cement has a certain amount of astringent action it is of the irritant quality, and the sensitive pulp tissues receive no assistance of an anodyne character, such as would assist it to recuperate from the initial shock, or the physical and chemical reaction which takes place at the time of application.

In conclusion, I would say that it seems reasonable for us to infer from this study that the conservation of exposed pulp applications of oxyphosphate of zinc cements should never be made to direct exposures, or even where there is a near approach to an exposure, and that first-class materials should always be employed and so mixed as to obtain complete chemical union of their elements, being especially careful to avoid an excess of free acid, and that much may be done to prevent the corrosive action of the phosphoric acid by a judicious application of an insulating varnish upon the dentin previous to the application of the cement.

### DISCUSSION.

In continuation of the discussion of the question of the influence of oxyphosphate fillings, especially on exposed or nearly exposed pulps, the following extracts from the January and February *Items of Interest* will be interesting. They were contributed by their authors in response to inquiries propounded by the editor of that journal to prominent teachers and practitioners throughout the country. While the replies are not at all conclusive, they indicate a nearly equal division of opinion on the question as to whether oxyphosphate cements devitalize the pulp when placed directly upon it or very near to it. Of seventeen replies, nine seem to favor the statement and eight believe otherwise.

Prof. Edward C. Kirk: I am quite familiar with the claim, so often made, that an oxyphosphate filling placed in a deep cavity will act as an irritant to a healthy pulp, and may eventually bring about the death of that organ. I am quite willing to admit the possibility of such a result, but I am not so clear as to the essential cause—namely, that pulp irritation and death in these cases is more likely to occur under an oxyphosphate filling than under any other kind of a filling in cavities of the same relative proximity to the pulp. In fact, I am largely of the opinion that death of the pulp in these cases is due to the near approach of caries to the pulp, rather than to chemical irritation from the oxyphosphate filling, or, as otherwise expressed, pulp death would be as likely to occur under any filling where the pulp was as nearly approached. Oxyphosphate has a questionable record in these cases, because it is usually resorted to as a filling material only in cases of deep-seated caries, and the record of pulp deaths in such cases would necessarily be much larger than in cavities suitable for treatment by metallic fillings.

It is true that two possible sources of irritation from oxyphosphate are to be considered apart from its physical properties, namely, those arising from the irritating character of certain of its component materials. These are free phosphoric acid and arsenic.

It is a well-known fact that chemical union of the powder (zinc oxide) and the liquid (one of the various modifications of tri-basic phosphoric acid) does not take place immediately, and is complete only after the lapse of a considerable period of time, often a number of hours, and until absolute union between acid and powder has taken place, there is always some free acid in the mass.

Proof of the imperfect nature of the chemical combination in the early stages of the mix may be found by two simple tests: First, the physical one of the dynamometer, which will show for a number of hours a gradually increasing resistance to crushing stress. Secondly, if before chemical combination is complete, and during the period when resistance to stress is rising to a maximum, the mix or pellet be placed upon the surface of moistened blue litmus paper, a distinctly acid reaction will be manifested by the formation of a red spot at the point of contact. The phosphoric acid liquid is in a highly concentrated solution, which is markedly hygroscopic, which latter quality every dentist knows by experience of the manner in which his bottle of cement liquid will abstract the moisture from the atmosphere during the damp weather of the summer and early autumn. With these properties of the acid in mind, it is quite conceivable that the avidity for moisture of the uncombined acid in a newly-placed oxyphosphate filling might lead to its osmosis in the dentinal structure, causing disintegration of the dentin and possible irritation of the pulp in cases of near approach to that organ.

I have alluded to the occurrence of arsenic in the cement powder. It is likewise a well-known fact that nearly all commercial preparations of zinc oxide contain slight quantities of arsenic, this latter element being constantly associated with zinc ores in nature, very few zinc-producing regions being free from it.

Dr. Vernon Hall has recently drawn attention to the common occurrence of arsenic in cement powders, but Dr. W. V. B. Ames, of Chicago, has, in commenting upon Dr. Hall's observations, drawn attention to an important fact in relation thereto, namely, that, under the circumstances governing the manufacture of cement powders, viz., the high heat to which they are subjected in the calcination process, the arsenic enters into combination with the zinc, forming zinc arsenite, rendering the arsenic fully inert, just as arsenic when swallowed is rendered inert by the formation of iron arsenite, by the administration of freshly prepared moist ferric oxide as an antidote.

Under these circumstances, I am inclined to the belief that the presence of arsenic discoverable in minute quantities in cement powders may be disregarded so far as any deleterious effect upon dentin or the pulp is concerned where such powders form the basis of zinc oxyphosphate fillings.

Prof. J. Taft: The pulp can not be affected by the phosphoric acid, unless it penetrates the covering of the pulp. The readiness with which this may be done would depend upon the thickness of the covering and the presence of free phosphoric acid.

After the mass has undergone the hardening process, there is not, if proper precautions have been used, enough free phosphoric acid to cause any injury.

The susceptibility of the pulp to irritation is a feature that must not be overlooked. In cases of good constitutional health and vigor, the pulp resists irritants in a marked degree, while in others it is very easily irritated. Pulps are sometimes exposed through almost imperceptible openings, through small projections from the pulp chamber. Great care should be exercised that this condition does not exist in any case. In treating cases where there is a suspicion of a liability to disease, most thorough examination should be made, and a thorough knowledge of the condition attained. The density of the dentin should be taken into account. In many cases it is very dense and resistant, and in others lacking this quality in a marked degree. If the pulp shows irritation before filling, an intervening substance may be used, as a thin layer of gutta-percha or Hill's stopping, or a solution of either may be used as a varnish. This, or some similar procedure, will serve as a good protection. There is, perhaps, no department of dental practice where there is more faulty work than in the management of exposed pulps, or those not properly protected by a substantial covering of the normal tissues.

The fact should not be overlooked that, in a great many susceptible cases, the pulp becomes affected early after the attack of decay, which has penetrated the dentin a considerable distance. A thorough knowledge of the subject, together with sharp discrimination, should be exercised in the cases where the welfare of the pulp comes in question.

Prof. George Edwin Hunt: I regret that I am compelled to preface my remarks with the admission that my knowledge on this subject is purely empirical. We have all had our clinical experiences, and it is from these that I draw my theories and deduct my conclusions.

While it can not be denied that some of the reported dead pulps under the conditions cited were either so diseased, or else so closely approached, that they would have died under any sort of filling, yet to my mind it seems clear that neither previous pathological conditions nor the proximity of a substance so non-irritating and so unsusceptible to thermal changes as a completed oxyphosphate cement filling will account for all of the cases met with in practice.

By a completed oxyphosphate filling, I mean one in which crystallization of the molecules has been perfected, and in which the chemical action incidental to "setting" has ceased. In my opinion, a completed oxyphosphate cement filling is non-irritant. Any deleterious action which a cement filling may have on the pulp tissue takes place before this chemical action ceases.

The liquid in the cement is the disturbing factor. Some cement liquids are more acid, and, I believe, more irritant than others. While I have never made the experiment, I will venture the assertion that glacial phosphoric acid in juxtaposition with the living pulp will create serious inflammation. If this be true, the phosphoric acid in the plastic mass will irritate the pulp in direct proportion to the quantity present. As it is necessary to use the cement in an uncompleted or plastic form, it is impossible to avoid this untoward effect.

In this connection a thought occurs to me which might serve as a basis for an investigation. It is a well-known scientific fact that a certain quantity of the liquid is necessary to properly affect a given quantity of the zinc oxide. In other words, the molecules of the powder and of the liquid combine in a definite ratio. Since this is so, if an excess of liquid is used, what becomes of it? Is completion of the filling delayed until this excess has been slowly given off, which, by penetration, may affect the pulp? How long does chemical action continue in the cement if an excess of the liquid be used? It takes something more than theorizing to answer these questions, but when the truth is known it will not surprise me to learn that, in many cases, the chemical action incidental to the completion of the filling is of some days' or perhaps weeks' duration, and that, in the interval, minute quantities of free phosphoric acid are being driven off. During this time these will produce a low grade of irritation highly calculated to depreciate the vitality of the delicate pulp tissue to the point of cessation of function. And the closer the cement approaches to the pulp the greater the certainty of the result and the quicker it will be accomplished.

Prof. G. V. Black: I have no sympathy with the opinion so widely held that oxyphosphate tends to destroy the tooth's pulp. I have had too much experience in capping pulps with oxyphosphate of zinc to think so, and have found that they live too well under this as a capping for me to suppose for one moment that there is anything in the oxyphosphate that is intrinsically obnoxious to the vitality of the pulp. I recognize that many pulps do die under oxyphosphate under conditions illy chosen for capping.

Prof. John T. Hart: I most firmly believe that an oxyphosphate filling in a deep cavity carries danger to the pulp. My supposition is that the free acid (phosphoric) on the surface of the filling, during the interval between its placement and crystallization, is absorbed into the tubules and stimulates the pulp; some pulps react under the stimulus favorably, others become so congested that they strangulate by pressure at the apical foramen.

Prof. G. S. Junkerman: That pulps die under oxyphosphate can not be denied. That some have died under oxyphosphate that would have survived some other kind of filling material is also not debatable; but I do not believe that in a perfectly typical case of a thin, perfect stratum of dentin being covered with a perfectly mixed cement, death of the pulp will occur.

There are many circumstances that alter the effect of cement used in deep-seated caries. Many times from imperfect diagnosis, due either to the carelessness of the operator, or often to our inability to determine whether there is or is not an actual exposure of the pulp, the results will prove unsatisfactory. If an imperceptible exposure exists, oxyphosphate will accomplish destruction of the pulp as readily as any other material placed upon it. Thus I do not believe that any pulp that is actually exposed can be preserved alive for any length of time under any kind of filling material.

I do not believe, however, that oxyphosphate should receive the censure that such results receive, where deep-seated caries has advanced to an actual exposure of the pulp, whether that exposure be perceptible or not. Many practitioners jump at conclusions exactly in cases of such a nature. If a perfect stratum of dentin exists between the pulp and all probability of actual exposure is removed, and if the cement is properly mixed, I do not believe that such a pulp will be injured. Take three typical cases in which a perfect stratum of dentin exists. Introduce in the first case the cement properly mixed, free from all superfluous acid. In the second case varnish with shellac the intervening stratum, as a precautionary measure, and introduce perfectly mixed cement. In the third case introduce cement that is not properly mixed, upon either a varished or unvarnished surface, there being a surplus of acid present; the first two cases will be successful, the last will prove fatal as to the vitality of the pulp.

The question is asked, How do cement fillings destroy pulps? I would answer this question by stating that, in my judgment, pulps are destroyed by the acid from the cement acting as an irritant to the pulp. We have no doubt established the fact that fluids permeate the dental tubuli, and that when there is a change in the chemical reaction there also occurs an osmosis outward or inward. The natural condition of the pulp is to be of an alkaline reaction. When you place fluid on the other side of the dental tubuli you produce an inward osmosis, and I feel thoroughly convinced that should the conditions of the pulp chamber be examined in a case where there has been a surplus of acid placed over the pulp, we would find phosphoric acid within the pulp chamber. The death of the pulp is accomplished no doubt from the effects of the phosphoric acid as an irritant, and the endeavors and struggles of the pulp to throw off this irritant, as would occur in any other part of the human body where an irritant were present.

Prof. Henry W. Morgan: I have often met with cases of facial neuralgia and abscesses upon the roots of teeth that had oxyphosphate fillings in them, and because I have had no other reasons to assign the conditions to, I have attributed the death of the pulp to the presence of a large mass of oxyphosphate zinc. I have believed it to be due to the presence of phosphoric acid.

The mixture of oxyphosphate is a chemical process that is not well understood, and in the hands of most men very carelessly handled. In many attempts to fill teeth with it there is an excess of phosphoric acid, which, I think, explains the devitalized condition of the pulp.

Prof. L. L. Dunbar: I believe any filling material in a deep cavity carries danger to the pulp in proportion to the conductivity of the material; this is so easily obviated, however, that the use of oxyphosphate need not be interdicted in such situations.

I am of the opinion that death of the pulp is to be accounted for by the careless use of large masses of oxyphosphate in cavities where some adverse impression has already been received by the pulp.

There is also a disposition upon the part of most practitioners to take for granted the statements of manufacturers in respect to the "non-irritating and non-conducting" qualities claimed for oxyphosphate, and to use them without due precaution, particularly in pulp conservative efforts.

I do not believe in the persistent action of oxyphosphate upon the organic elements of a tooth, but think that the deleterious effect is one that would occur under any filling material used in the same way, the trouble being that we expect too much from oxyphosphate.

Dr. E. W. Wedelstaedt: I believe an oxyphosphate filling in a deep cavity does very often carry danger to the pulp. Why? For the simple reason that in a great majority of cases, the filling is placed on a whole mass of decalcified dentin. Just so long as our text-books do not condemn the leaving of soft decay in cavities, and just so long as some teachers will teach doctrines of this kind, just so long will any filling placed on this mass be the means of ultimately destroying the pulp. When cement or any filling is placed on a mass of decay in a cavity, the micro-organisms are shut off from the air and their entire environment changed. They get old, die and their solubility readily takes place. After resolution, the cell contents is simply a toxalbumen. This is the same as any other toxine—capable of destroying life. The micro-organisms have penetrated just about so far into the tooth. When the toxalbumen is liberated the fibrils, which are in the immediate vicinity, are first affected by this poison. They absorb it and carry it on to the main body of the pulp, which in turn is poisoned the same as were the fibrils. Whether or not this is the exact process, I can not state. A number of years ago Dr. Black called my attention to this matter, and from that time to the present day I have carefully studied this subject of filling over decay, and I have some definite idea of what the results are, for I have seen them many times. I do not believe that the cement, in some cases, has any more to do with the destruction of the pulp than has any other filling material that might be placed in that cavity, unless there were free phosphoric acid in the cement, in which case it might possibly destroy the micro-organisms in the immediate vicinity more quickly than if a metal filling had been used.

Phosphoric acid is caustic, and when it is mixed with the oxide of zinc, it still retains a portion of its causticity. I did not believe that until a minute ago. I picked up a piece of cement filling that I had mixed about two months ago, cracked it in the dynamometer and broke the pieces between my teeth. I have those pieces in my mouth, and each time I break a piece there is a fresh acid taste. If I can taste this acid so distinctly, surely the tooth fibrils must be effected by it.

